# QuickStats

**Published:** 2014-01-31

**Authors:** FROM THE NATIONAL CENTER FOR HEALTH STATISTICS

**Figure:**
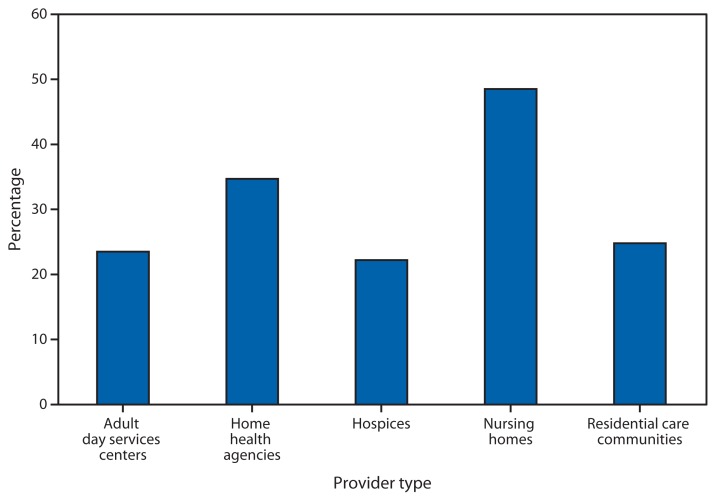
Percentage of Users^*^ of Long-Term Care Services with a Diagnosis of Depression,^†^ by Provider Type — National Study of Long-Term Care Providers, United States, 2011 and 2012 ^*^ Denominators used to calculate percentages for adult day services centers, nursing homes, and residential care communities were derived from the number of residents/participants on a given day in 2012. Denominators used to calculate percentages for home health agencies and hospices were the number of patients whose episode of care in a home health agency ended at any time in 2011, and the number of patients who received care from Medicare-certified hospices at any time in 2011. ^†^Participating administrators and directors of residential care communities and adult day services centers were asked, “Of the residents currently living at this community/participants enrolled at this center, about how many have been diagnosed with depression?”

In 2011 and 2012, the percentage of users of long-term care services with a diagnosis of depression was highest in nursing homes (49%) and home health agencies (35%), and lowest in residential care communities (25%), adult day services centers (24%), and hospices (22%). The percentage of users with a diagnosis of depression in nursing homes (49%) was approximately twice that of those in adult day services centers (24%) or residential care communities (25%) in 2012.

**Source:** Harris-Kojetin L, Sengupta M, Park-Lee E, Valverde R. Long-term care services in the United States: 2013 overview. Hyattsville, MD: US Department of Health and Human Services, CDC; 2013. Available at http://www.cdc.gov/nchs/data/nsltcp/long_term_care_services_2013.pdf.

**Reported by:** Vincent Rome, MPH, vrome@cdc.gov, 301-458-4466; Manisha Sengupta, PhD; Lauren Harris-Kojetin, PhD; Eunice Park-Lee, PhD; Roberto Valverde, MPH.

